# Effectiveness of an Out-of-Pocket Cost Removal Intervention on Health Check Attendance in Japan

**DOI:** 10.3390/ijerph18115612

**Published:** 2021-05-24

**Authors:** Hiroshi Murayama, Yuta Takahashi, Setaro Shimada

**Affiliations:** 1 Research Team for Social Participation and Community Health, Tokyo Metropolitan Institute of Gerontology, Tokyo 173-0015, Japan; 2 Health and Welfare Bureau, City of Yokohama, Kanagawa 231-0005, Japan; yu52-takahashi@city.yokohama.jp (Y.T.); se00-shimada@city.yokohama.jp (S.S.)

**Keywords:** health check, attendance, economic intervention, out-of-pocket cost removal, Japan

## Abstract

Annual health checks are important for identifying individuals at high risk for cardiometabolic diseases. However, there are socioeconomic disparities in health check attendance rates, and an intervention to lower financial barriers could be useful for increasing health check utilization. In this study, we aimed to evaluate the effectiveness of an out-of-pocket cost removal intervention on health check attendance in Japan. Data were obtained on beneficiaries of the National Health Insurance system of Yokohama City, Kanagawa Prefecture, Japan. In 2018, Yokohama started an intervention to remove out-of-pocket costs for specific health checks for all National Health Insurance beneficiaries. We analyzed data from 2015−2018 (131,295 people aged 40–74 years; 377,660 observations). A generalized estimating equation showed that people were more likely to receive specific health checks in 2018 (after the out-of-pocket cost removal intervention started) than in 2017 (immediately before the intervention; odds ratio [95% confidence interval] = 1.167 [1.149–1.185]), after adjusting for age, gender, tax exemption, and residential area. Stratified analyses revealed that the effectiveness of the out-of-pocket cost removal intervention was greater among the older age group and those who did not receive a tax exemption (i.e., those with relatively higher income). The present study showed that the out-of-pocket cost removal intervention could promote specific health check utilization. This indicates that removing financial barriers could motivate people’s behavior regarding health check attendance.

## 1. Introduction

Cardiometabolic diseases (CMDs), including cardiovascular disease, stroke, diabetes, and chronic kidney disease, remain the most common causes of death worldwide [1]. CMDs are primarily caused by metabolic syndrome, which is a cluster of metabolic disorders. The underlying causes of metabolic syndrome include obesity, physical inactivity, and older age [2]; thus, with increasing rates of obesity [3] and insufficient physical activity [4], in combination with the global aging population, there is an urgent need for screening of CMD risks and prevention strategies. In several developed countries, general health checks are conducted to prevent CMDs [5]. A study using a quasi-experimental design reported that such screening reduced cardiometabolic risks like abdominal obesity and waist circumferences [6], although meta-analyses revealed that general health checks were unlikely to reduce mortality and morbidity risks [7,8]. This implies that general health checks seem beneficial to decrease CMD risks. For such population-based prevention efforts to succeed, increasing the rates of health check participation is essential. 

According to Andersen’s health care utilization model, health care use is principally influenced by predisposing, enabling, and need factors [9,10]. Predisposing factors primarily influence the use of health services indirectly and consist of sociodemographic characteristics. Enabling factors encompass individual and contextual resources which facilitate or impede the use of health services, such as availability of resources (e.g., income), availability, and access to the service. Need factors directly motivate health services use, such as health conditions (e.g., illness or disease). A systematic review reported that many studies identified financial condition as one of the most critical determinants of health check attendance [11]. Therefore, economic interventions to encourage attending health checks can be an enabling factor. 

Meta-analyses illustrated that approaches that provide economic intervention, including removal of financial barriers and financial incentives, were practical for increasing health check attendance [12,13]; in particular, removal of financial barriers had a greater effect on attendance. For instance, a study in Denmark reported that the rate of preventive health check attendance was higher in areas where the checks were free than in areas where a payment was required (40 US dollars to undergo health checks) [14]. These findings indicate that interventions to remove or reduce costs can promote health check attendance. This corresponds with the concept of Universal Health Coverage, in which all individuals are assured access to health services without the financial burden, and the Sustainable Development Goals (Goal 3: Ensure healthy lives and promote well-being for all at all ages). However, there is still sparse evidence on the effectiveness of economic interventions to increase health check attendance in non-Western nations, as earlier studies came from Western nations. Although the aforementioned meta-analyses revealing the effectiveness of cost removal interventions included two papers [12] and nine papers [13], respectively, no studies were derived from non-Western countries. As attitudes toward health, which are determinants of health check attendance [11], can be influenced by individual cultural backgrounds, intervention effects might differ across regions. Thus, further research conducted in non-Western populations (e.g., Asian populations) is warranted.

In 2008, Japan established a national health policy to prevent lifestyle-related diseases such as CMDs. This comprehensive preventive policy involves nationwide screening (called specific health checks, or *Tokutei Kenshin* in Japanese). Annual health checks focused on CMDs are conducted for individuals and their family members aged 40–74 years and covered by the primary beneficiaries under Japan’s health insurance system. Out-of-pocket (OOP) costs for specific health checks depend on the health insurance society in which people are enrolled.

Socioeconomic disparities between those who do and do not utilize health checks have been observed in Japan, similar to those in Western countries. Lower income, less financial stability, and higher unemployment were found to be associated with a lower likelihood of health check participation [15,16]. Therefore, removing OOP costs, which can lower the financial barrier, could be useful for increasing health check attendance rates. Although previous research reported the effectiveness of an OOP cost removal intervention on cancer screening utilization in Japan [17,18], no study to date has examined whether this would also help increase health check attendance. Therefore, this study aimed to evaluate the effectiveness of an OOP cost removal intervention on health check attendance in Japan. 

## 2. Materials and Methods

### 2.1. Sample and Procedures

In the present study, data were obtained from the National Health Insurance (NHI) system of Yokohama City, Kanagawa Prefecture, Japan. Yokohama is the capital of Kanagawa Prefecture, located 30 km southwest of central Tokyo [19]. As of January 2018, Yokohama was reported to have 18 administrative wards and a population of 3,733,084. At the time of this study, the NHI covered approximately 20% of the total city population. 

Japan has more than 3000 insurers, which can generally be divided into three types: residence-based health insurance (i.e., NHI), occupation-based health insurance, and health insurance for people aged ≥ 75 years. All Japanese individuals are covered by a health insurance system, except recipients of public assistance (*seikatsu-hogo* in Japanese; approximately 1.6% of the total Japanese population in 2019). The NHI is a system for farmers, self-employed individuals, pensioners, and their dependents who are aged ≤74 years, and is administered by local municipalities. The specific health checks for the NHI beneficiaries are mainly conducted at hospitals and medical examination facilities in the community. The NHI beneficiaries are notified annually to attend specific health checks by local municipalities, and they apply for the health checks themselves. 

In 2018, Yokohama began an intervention that eliminated OOP costs for specific health checks for all NHI beneficiaries aged 40–74 years. Before 2018, OOP costs for specific health checks in Yokohama depended on an individual’s annual income level. In Japan, people with a total annual income below a certain threshold do not need to pay city inhabitant taxes; thus, receiving a tax exemption indicates one has a relatively lower income. Prior to 2018, specific health checks in Yokohama cost 1200 yen (approximately 11 US dollars) and 400 yen (approximately 4 US dollars), among those without and with tax-exempt status, respectively. For reference, the beneficiaries of occupation-based health insurance (aged 40–74 years) also have to pay OOP costs for specific health checks, but the cost depends on the insurers.

To examine the influence of the OOP cost removal intervention, we analyzed four years of panel data (2015–2018). Moreover, to consider the effect of residents’ health condition on the intervention’s outcome, we selected 3 out of 18 administrative wards based on the standardized mortality ratio of the area in 2017 (Aoba [0.82; 1st out of 18], Kanazawa [0.97; 9th out of 18], and Seya [1.04; 13th out of 18]) and the proportion of those who had CMD risks in the specific health checks from the area in 2016 (Aoba [low], Kanazawa [middle], and Seya [high]) [20]. These variations in residents’ health conditions by the residential area can be regarded as the need factor in Andersen’s health care utilization model. In addition, these three areas had different socioeconomic levels (i.e., residents’ average income level) in 2018: Aoba (1st out of 18), Kanazawa (9th out of 18), and Seya (18th out of 18). Consequently, we analyzed all NHI beneficiaries aged 40–74 years who lived in those three wards in April (the beginning of the fiscal year in Japan) of each year. Those who were hospitalized or admitted to care facilities were unlikely to participate in specific health checks because their health conditions were followed by health professions at the hospitals or care facilities. Therefore, we excluded them from the analytic sample. A total of 131,295 people with 377,660 observations (100,023, 96,985, 92,426, and 88,226, in 2015, 2016, 2017, and 2018, respectively) were included. 

The study protocol was reviewed and approved by the Research Ethics Committee of the University of Tokyo (No. 19-399; approved on 26 February 2020). The data were anonymized before analysis by officers of Yokohama, who did not participate in data analysis. All personally identifiable information was completely removed from the dataset. 

### 2.2. Measures

We set specific health check utilization as the outcome variable in the present study. This was defined as whether a person received a specific health check in each observational year (i.e., 2015–2018). The observational year was used as the exposure variable, with 2017 set as the reference. This variable represents whether the intervention was performed (i.e., 2015–2017 were the years before the OOP cost removal intervention started, and 2018 was the year the intervention was implemented), and is regarded as an enabling factor in Andersen’s health care utilization model. 

Age (40–49, 50–59, 60–69, or 70–74 years), gender (male or female), tax exemption (receiving or not receiving), and residential area (Aoba, Kanazawa, or Seya) were used as covariates in the analysis. Age and gender can be considered as predisposing factors, and tax exemption as an enabling factor. As mentioned above, we considered the residential area (i.e., a proxy of residents’ health condition) as a need factor. Tax-exempt status was used as a proxy of annual income, as receiving a tax exemption would indicate having a relatively lower annual income. When participants had no taxable annual income at least once during the observational period, we categorized them as receiving a tax exemption. 

### 2.3. Statistical Analysis

Observational data is nested within a participant individual. Therefore, a generalized estimating equation that accounted for the extra component of variation within participants was used to adjust for correlations among repeated measures.

To assess whether the likelihood to attend the health check differed by year (particularly in 2018, when the OOP cost removal intervention was started), we added the year term in the generalized estimating equation (Model 1). In Model 2, we additionally controlled for participant characteristics (i.e., age, gender, tax exemption, and residential area). In Model 3, interaction terms between each covariate and observational year (i.e., age × year, gender × year, tax exemption × year, and residential area × year) were examined to determine whether the effectiveness of the OOP cost removal intervention on specific health checks utilization differed by participant characteristics and residential area. When any significant interaction was detected, we performed a stratified analysis by participant characteristics to identify the differences in effectiveness by factor. 

Furthermore, to control for variation by residential area, we used other statistical approaches in addition to simple statistical adjustment in the model: area-stratified analyses and multilevel logistic regression analyses (three-level: observation, participant individual, and residential area). Odds ratios (ORs) with 95% confidence intervals (CIs) were calculated. Statistical analyses were performed using SAS version 9.4 (SAS Institute Inc., Cary, IL, USA).

## 3. Results

Table 1 shows participant characteristics at baseline. When participants had multiple observations during the four years, we used each baseline information. The average age was 61.2 years (standard deviation, 10.4), and the largest age group ranged from 60–69 years (40.4%). Of the participants, 44.8% were male and 44.5% received a tax exemption during the observational period. People who attended a specific health check tended to be older, female, and not receive tax exemption. 

Table 2 indicates baseline participant characteristics by residential area. Participants residing in Kanazawa were likely older than those in the other areas. The proportion of those with tax exemption was the highest in Seya and the lowest in Aoba. 

Table 3 presents the association between the OOP cost removal intervention and specific health check utilization. In Model 1, people were more likely to receive specific health checks in 2018 (after the OOP cost removal intervention began) than in 2017 (immediately before the intervention started; OR [95% CI] = 1.180 [1.163–1.198]), while the likelihood of receiving specific health checks in 2015 and 2016 was almost the same as that in 2017 (1.009 [0.993–1.025] in 2015, and 0.950 [0.936–0.964] in 2016). In Model 2, even after adjusting for age, gender, tax exemption, and residential area, the associations remained unchanged. The likelihood of receiving specific health checks increased by approximately 17% in 2018, compared to 2017 (1.167 [1.149–1.185]), and there was a statistically significant, but relatively small difference in likelihood from 2015–2017 (1.025 [1.008–1.042] in 2015 and 0.959 [0.945–0.974] in 2016). Regarding covariates, younger age, gender (male), receiving a tax exemption, and Seya residency were associated with a lower likelihood of specific health check attendance. 

The interactions between the covariates and observational years were added in Model 3. We found significant interactions between age subgroups and the year 2018 (0.923 [0.865–0.986], 0.943 [0.891–0.998], and 0.953 [0.917–0.990] for 40–49, 50–59, and 60–69 years, respectively). We also found a significant interaction between receiving a tax exemption and the year 2018 (0.936 [0.905–0.968]). 

Table 4 shows the analyses conducted by residential area, using the same modeling as Table 3. The result was consistent with that among the total sample: people tended to receive more specific health checks in 2018 than in 2017, while the likelihoods of receiving specific health checks (i.e., ORs) were similar from 2015–2017. In addition, the trend of statistical significance on the interaction was also similar to that in the total sample (shown in Tables S1–S3 in the Supplementary Materials). 

We ran a three-level multilevel logistic regression analysis to confirm consistency of the results across the analytic approach. The results of the multilevel analysis were also similar to those in Table 3. 

To better understand the interactions, we performed stratified analyses by age and tax exemption, as shown in Table 5 and Table 6. The likelihood of utilizing specific health checks was higher among the older age group in 2018 than in 2017 (1.113 [1.048–1.182], 1.141 [1.085–1.201], 1.146 [1.118–1.174], and 1.206 [1.175–1.237] for 40–49, 50–59, 60–69, and 70–74 years, respectively). In addition, compared to 2017, the likelihood of utilizing specific health checks in 2018 was approximately 21% higher among those without a tax exemption (1.207 [1.179–1.236]), while it was approximately 14% higher among those with a tax exemption (1.138 [1.116–1.161]). The trends of the associations were consistent with the total sample in each residential area. 

## 4. Discussion

Based on NHI data for beneficiaries aged 40–74 years, in this study, we examined the effectiveness of an OOP cost removal intervention on attendance rates for specific health checks to screen individuals at high risk for CMDs. It is important to promote access to health services for everyone regardless of their demographic and socioeconomic backgrounds; this has also been proposed by the Universal Health Coverage and the Sustainable Development Goals. To the best of our knowledge, this was the first study to evaluate the effectiveness of the intervention regarding the removal of financial barriers (i.e., the OOP cost removal) for increasing health check attendance in an Asian population. CMDs are a major cause of death worldwide, including in Asian countries. Therefore, it would be useful to determine whether economic interventions to remove financial barriers could increase the likelihood that individuals would utilize health checks.

We found that the OOP cost removal intervention examined in this study could increase specific health check utilization. Although the change in likelihood to receive the health checks from 2015 to 2017 was statistically significant; its effect sizes (i.e., ORs in 2015 and 2016) were relatively smaller than those between 2017 and 2018. The nationwide attendance rates of the specific health checks among the NHI beneficiaries in Japan were 36.3%, 36.6%, 37.2%, and 37.9% in 2015, 2016, 2017, and 2018, respectively. As there was no obvious increase from 2015 to 2018, we regarded that the increase of likelihood in health check attendance between 2017 and 2018 in this study was related to the OOP cost removal intervention. Previous systematic reviews on economic interventions to increase screenings for cardiovascular disease risk factors reported that removing financial barriers (e.g., offering free screening) increased attendance, while rewards or monetary incentives (e.g., providing shopping vouchers, gifts, or bus tickets) did not [12,13]. The results of the current study were consistent with these systematic reviews. 

We estimated that the likelihood of receiving a specific health check increased by approximately 17% following the OOP cost removal intervention among both the total sample and each area’s residents, but this estimate seemed to be relatively smaller than what was found in previous studies [14,21]. In this study, before the intervention, OOP costs were approximately 11 US dollars and 4 US dollars for people who did not and did receive a tax exemption, respectively, and these were fully covered by the subsidy in 2018. In contrast, in a study regarding a financial barrier removal intervention [14], a cost of 40 US dollars was fully covered in the intervention area for health examinations related to coronary heart disease, leading to a greater attendance rate increase than was found in the present study (66% vs. 37%). This can be partly explained by the price elasticity of demand, which refers to the relationship between price and quantity demanded: a greater amount of OOP cost removal would lead to a higher attendance. In addition, as the stratified analysis revealed that the effectiveness of the OOP cost removal intervention was greater among those without a tax exemption, utilization of a health check might be affected by income elasticity (the relation between income and demand) as well as price elasticity. However, it is known that demand of healthcare service, in general, tends to be price- and income-inelastic [22]. Further research should be conducted to investigate elasticity of health check demand in order to develop feasible policy schemes regarding health examination in the future.

Our age-stratified analyses showed that the effectiveness of the OOP cost removal intervention was stronger in the older subgroup. Compared with younger people, older people tend to perceive health checks as being more relevant to their lives and are more ready to face the related outcomes, which are factors known to facilitate health check utilization [23,24]. The OOP cost removal could encourage behaviors among older individuals ready to attend health checks, as compared with younger individuals who may be less ready. 

Earlier studies revealed that higher income and older age were associated with greater health check attendance [25,26,27,28]. Our results also indicated that the OOP cost removal intervention promoted health check attendance among older people and those with a relatively higher income. These findings indicate that it is possible that the intervention might widen socioeconomic disparities in health check access. Indeed, our data showed that the gap in attendance rate between those aged 40–49 and 70–74 years and between those with and without tax exemption widened after the intervention. Although this study generally showed that the OOP cost removal intervention could promote health check participation, when developing or evaluating such strategies using economic interventions, policymakers should consider the possibility that the impact of different sociodemographic factors on the effectiveness of these interventions could widen the disparities in health check access. 

The present study has some limitations. First, we did not adjust for several potential confounders in the analysis. We obtained the data from the NHI system; however, variables such as sociodemographic conditions and health status were not available in the present study. Furthermore, individual perceptions, such as relevance of health checks, attitudes toward disease prevention, and expectations about health check outcomes, could affect health check utilization [11,23,24,29]. These factors should be considered in future analyses. Second, we only evaluated the immediate effects of the OOP cost removal intervention. Thus, a longer follow-up period would be necessary to monitor the sustainability of the intervention’s effectiveness. Third, there might be other factors increasing the attendance rate of the health checks, other than the OOP cost removal intervention. Due to data unavailability, we could not set the control group (e.g., the NHI beneficiaries in another municipality). However, only a gentle increase in attendance rate was observed during the period of 2015–2018 in Japan; we considered that the increase of likelihood of health check attendance in our sample was related to the intervention. Fourth, in this study, we could not follow up on the possible behavioral changes, reductions in the cardiometabolic risks, and adverse health outcomes (e.g., morbidity and mortality) among the participants. In particular, as we found that individuals who were older and had a relatively higher income were the most sensitive to the OOP cost removal intervention, it is important to explore whether the health checks were beneficial for them. Fifth, because this study was performed using the data of the NHI beneficiaries in a local municipality, care should be taken when generalizing the findings. As the OOP cost of the health checks differs by health insurers, our findings might not be generalizable to other health insurers, but possibly to NHI beneficiaries in other municipalities. In addition, because we excluded those hospitalized or admitted to care facilities from the sample, our findings can be generalized mainly to community-dwelling people. 

## 5. Conclusions

The present study showed that an OOP cost removal intervention could promote specific health check attendance among NHI beneficiaries between the ages of 40–74 years in Japan. This indicates that this type of economic intervention (i.e., removal of financial barriers) could motivate people to utilize health checks. In addition, this effect was greater among the older age group and people with relatively higher income, suggesting that the effectiveness of the financial barrier removal intervention might differ according to sociodemographic characteristics. Policymakers should, therefore, consider that removing OOP costs could potentially widen the disparities in health check access.

## Figures and Tables

**Table 1:** Participant characteristics at baseline (*N* = 131,295).

Variable	Category	Total Sample	Attending a Specific Health Check (*n* = 27,385; 20.9%)	Not Attending a Specific Health Check (*n* = 103,910; 79.1%)
		Mean ± SD or %	Mean ± SD or %	Mean ± SD or %
Age (years)		61.2 ± 10.4	61.3 ± 8.9	60.3 ± 10.6
	40–49 years	19.7	10.4	22.1
	50–59 years	15.6	11.0	16.8
	60–69 years	40.4	46.8	38.7
	70–74 years	24.3	31.8	22.4
Gender				
	Male	44.8	39.7	46.1
	Female	55.2	60.3	53.9
Tax exemption				
	Receiving	44.5	43.3	44.8
	Not receiving	55.5	56.7	55.2
Residential area				
	Aoba	43.2	43.6	43.1
	Kanazawa	34.9	36.7	34.5
	Seya	21.9	19.7	22.5

SD: standard deviation.

**Table 2:** Participant characteristics at baseline by residential area (*N* = 131,295).

Variable	Category	Aoba	Kanazawa	Seya
		Mean ± SD or %	Mean ± SD or %	Mean ± SD or %
Age (years)		60.5 ± 10.6	62.2 ± 10.0	60.9 ± 10.6
	40–49 years	21.3	16.7	21.4
	50–59 years	17.3	13.6	15.4
	60–69 years	38.5	43.8	38.6
	70–74 years	22.8	26.0	24.7
Gender				
	Male	43.7	44.9	46.6
	Female	56.3	55.1	53.4
Tax exemption				
	Receiving	53.9	56.0	57.9
	Not receiving	46.1	44.0	42.1

SD: standard deviation.

**Table 3:** Increases in specific health check attendance after the out-of-pocket cost removal intervention in total sample.

Variable	Category	Attendance Rate	Model 1	Model 2	Model 3
			OR (95% CI)	OR (95% CI)	OR (95% CI)
Year	2015	21.2%	1.009 (0.993–1.025)	1.025 (1.008–1.042)	1.021 (1.003–1.039)
	2016	20.2%	0.950 (0.936–0.964)	0.959 (0.945–0.974)	0.960 (0.945–0.976)
	2017	21.1%	1.000	1.000	1.000
	2018	24.0%	1.180 (1.163–1.198)	1.167 (1.149–1.185)	1.158 (1.139–1.177)
Age	40–49 years			0.328 (0.314–0.342)	0.328 (0.315–0.342)
	50–59 years			0.448 (0.430–0.466)	0.448 (0.431–0.466)
	60–69 years			0.820 (0.800–0.841)	0.821 (0.800–0.842)
	70–74 years			1.000	1.000
Gender	Male			0.742 (0.721–0.763)	0.742 (0.721–0.763)
	Female			1.000	1.000
Tax exemption	Receiving			0.874 (0.850–0.899)	0.874 (0.850–0.899)
	Not receiving			1.000	1.000
Residential area	Aoba			1.000	1.000
	Kanazawa			0.986 (0.958–1.014)	0.986 (0.958–1.014)
	Seya			0.853 (0.825–0.882)	0.853 (0.824–0.882)
Interactions	40–49 years × year of 2015				0.943 (0.881–1.010)
	50–59 years × year of 2015				0.997 (0.937–1.060)
	60–69 years × year of 2015				0.996 (0.954–1.040)
	40–49 years × year of 2016				1.014 (0.951–1.081)
	50–59 years × year of 2016				0.982 (0.928–1.039)
	60–69 years × year of 2016				0.988 (0.951–1.027)
	40–49 years × year of 2018				0.923 (0.865–0.986)
	50–59 years × year of 2018				0.943 (0.891–0.998)
	60–69 years × year of 2018				0.953 (0.917–0.990)
	Male × year of 2015				1.000 (0.964–1.038)
	Male × year of 2016				0.987 (0.954–1.021)
	Male × year of 2018				1.000 (0.967–1.035)
	Receiving tax exemption × year of 2015				1.044 (1.006–1.082)
	Receiving tax exemption × year of 2016				1.018 (0.984–1.053)
	Receiving tax exemption × year of 2018				0.936 (0.905–0.968)
	Kanazawa × year of 2015				1.015 (0.979–1.053)
	Seya × year of 2015				1.032 (0.988–1.078)
	Kanazawa × year of 2016				1.024 (0.990–1.060)
	Seya × year of 2016				1.030 (0.989–1.073)
	Kanazawa × year of 2018				0.968 (0.935–1.001)
	Seya × year of 2018				0.987 (0.947–1.028)

CI: confidence interval. OR: odds ratio.

**Table 4:** Increases in specific health check attendance after the out-of-pocket cost removal intervention in subgroups by residential area.

Variable	Category	Attendance Rate	Model 1	Model 2	Model 3
			OR (95% CI)	OR (95% CI)	OR (95% CI)
Aoba					
Year	2015	21.4%	0.996 (0.972–1.020)	1.011 (0.986–1.037)	1.010 (0.984–1.037)
	2016	20.4%	0.938 (0.917–0.959)	0.945 (0.923–0.967)	0.949 (0.926–0.972)
	2017	21.4%	1.000	1.000	1.000
	2018	24.6%	1.198 (1.171–1.226)	1.184 (1.156–1.211)	1.178 (1.149–1.207)
Kanazawa					
Year	2015	22.3%	1.014 (0.988–1.040)	1.028 (1.001–1.056)	1.026 (0.996–1.057)
	2016	21.3%	0.957 (0.934–0.981)	0.967 (0.943–0.991)	0.967 (0.940–0.994)
	2017	22.1%	1.000	1.000	1.000
	2018	24.7%	1.158 (1.131–1.187)	1.150 (1.122–1.178)	1.132 (1.101–1.165)
					
Seya					
Year	2015	19.2%	1.034 (0.998–1.070)	1.048 (1.011–1.087)	1.038 (0.999–1.079)
	2016	18.2%	0.966 (0.935–0.998)	0.976 (0.944–1.010)	0.970 (0.936–1.006)
	2017	18.7%	1.000	1.000	1.000
	2018	21.3%	1.178 (1.140–1.218)	1.164 (1.125–1.204)	1.152 (1.111–1.196)

CI: confidence interval. OR: odds ratio. Model 1: unadjusted. Model 2: adjusted for age, gender, and tax exemption. Model 3: adjusted for age, gender, tax exemption, and interactions (between age and year, between gender and year, and between tax exemption and year).

**Table 5:** Stratified analysis by age for increases in specific health check attendance after the out-of-pocket cost removal intervention: total sample and subgroups by residential area.

Variable	Category	40–49 Years		50–59 Years		60–69 Years		70–74 Years	
		Attendance Rate	OR (95% CI)	Attendance Rate	OR (95% CI)	Attendance Rate	OR (95% CI)	Attendance Rate	OR (95% CI)
Total sample ^a^									
Year	2015	10.5%	0.974 (0.916–1.035)	14.6%	1.028 (0.975–1.085)	23.9%	1.029 (1.002–1.056)	27.3%	1.031 (1.000–1.063)
	2016	10.6%	0.977 (0.922–1.036)	13.6%	0.945 (0.899–0.993)	22.6%	0.953 (0.931–0.976)	26.0%	0.965 (0.939–0.991)
	2017	10.8%	1.000	14.2%	1.000	23.5%	1.000	26.7%	1.000
	2018	12.1%	1.113 (1.048–1.182)	15.9%	1.141 (1.085–1.201)	26.4%	1.146 (1.118–1.174)	30.6%	1.206 (1.175–1.237)
Aoba ^b^									
Year	2015	11.3%	0.959 (0.879–1.046)	15.3%	1.008 (0.935–1.087)	24.4%	1.036 (0.995–1.078)	27.8%	1.000 (0.953–1.049)
	2016	11.5%	0.984 (0.906–1.069)	14.5%	0.943 (0.880–1.011)	22.8%	0.949 (0.914–0.984)	26.3%	0.928 (0.890–0.969)
	2017	11.7%	1.000	15.2%	1.000	23.8%	1.000	27.8%	1.000
	2018	13.6%	1.136 (1.042–1.238)	17.6%	1.200 (1.118–1.287)	27.3%	1.179 (1.134–1.226)	31.4%	1.192 (1.146–1.241)
Kanazawa ^b^									
Year	2015	10.8%	1.002 (0.895–1.121)	15.2%	1.079 (0.980–1.188)	24.5%	0.993 (0.952–1.035)	27.7%	1.063 (1.012–1.117)
	2016	10.5%	0.971 (0.874–1.080)	13.9%	0.978 (0.892–1.072)	23.5%	0.942 (0.907–0.979)	26.3%	0.991 (0.948–1.035)
	2017	10.8%	1.000	14.1%	1.000	24.7%	1.000	26.5%	1.000
	2018	11.4%	1.069 (0.958–1.193)	15.4%	1.089 (0.991–1.196)	27.1%	1.116 (1.074–1.160)	30.4%	1.211 (1.161–1.263)
Seya ^b^									
Year	2015	8.8%	0.969 (0.848–1.106)	12.3%	1.007 (0.892–1.137)	22.0%	1.091 (1.028–1.158)	25.8%	1.038 (0.972–1.109)
	2016	8.8%	0.969 (0.853–1.100)	11.2%	0.904 (0.807–1.012)	20.4%	0.987 (0.935–1.041)	25.0%	0.991 (0.935–1.050)
	2017	9.1%	1.000	12.2%	1.000	20.6%	1.000	25.2%	1.000
	2018	10.1%	1.122 (0.981–1.283)	13.1%	1.076 (0.962–1.204)	23.3%	1.135 (1.072–1.202)	29.3%	1.221 (1.153–1.292)

CI: confidence interval. OR: odds ratio. ^a^: Adjusted for gender, tax exemption, and residential area. ^b^: Adjusted for gender and tax exemption.

**Table 6:** Stratified analysis by tax exemption status for increases in specific health check attendance after the out-of-pocket cost removal intervention: total sample and subgroups by residential area.

Variable	Category	Receiving Tax Exemption		Not Receiving Tax Exemption	
		Attendance Rate	OR (95% CI)	Attendance Rate	OR (95% CI)
Total sample ^a^					
Year	2015	21.6%	1.039 (1.017–1.061)	20.7%	1.001 (0.976–1.027)
	2016	20.3%	0.961 (0.942–0.980)	20.1%	0.954 (0.932–0.977)
	2017	21.0%	1.000	21.1%	1.000
	2018	23.4%	1.138 (1.116–1.161)	25.1%	1.207 (1.179–1.236)
Aoba ^b^					
Year	2015	22.1%	1.027 (0.994–1.062)	20.5%	0.985 (0.948–1.024)
	2016	20.7%	0.943 (0.915–0.973)	20.0%	0.945 (0.912–0.979)
	2017	21.7%	1.000	21.1%	1.000
	2018	24.3%	1.145 (1.110–1.180)	25.5%	1.238 (1.195–1.284)
Kanazawa ^b^					
Year	2015	22.3%	1.031 (0.996–1.067)	22.4%	1.018 (0.977–1.061)
	2016	21.1%	0.964 (0.933–0.996)	21.7%	0.967 (0.931–1.006)
	2017	21.8%	1.000	22.6%	1.000
	2018	24.1%	1.134 (1.098–1.171)	26.0%	1.172 (1.128–1.217)
Seya ^b^					
Year	2015	19.7%	1.075 (1.027–1.125)	18.4%	1.009 (0.952–1.070)
	2016	18.4%	0.991 (0.949–1.034)	17.8%	0.954 (0.905–1.007)
	2017	18.6%	1.000	18.8%	1.000
	2018	20.8%	1.135 (1.086–1.186)	22.4%	1.208 (1.145–1.274)

CI: confidence interval. OR: odds ratio. ^a^: Adjusted for age, gender, and residential area. ^b^: Adjusted for age and gender.

## Data Availability

The datasets used and analyzed during the current study are available from the corresponding author on reasonable request.

## Supplementary Materials

The following are available online at https://www.mdpi.com/article/10.3390/ijerph18115612/s1, Table S1: Increases in specific health check attendance after the out-of-pocket cost removal intervention in Aoba, Table S2: Increases in specific health check attendance after the out-of-pocket cost removal intervention in Kanazawa, Table S3: Increases in specific health check attendance after the out-of-pocket cost removal intervention in Seya.

## References

[1] GBD 2017 Causes of Death Collaborators (2018). Global, regional, and national age-sex-specific mortality for 282 causes of death in 195 countries and territories, 1980-2017: A systematic analysis for the Global Burden of Disease Study 2017. Lancet.

[2] Park Y.W., Zhu S., Palaniappan L., Heshka S., Carnethon M.R., Heymsfield S.B. (2003). The metabolic syndrome: Prevalence and associated risk factor findings in the US population from the Third National Health and Nutrition Examination Survey, 1988-1994. Arch. Intern. Med..

[3] Blüher M. (2019). Obesity: Global epidemiology and pathogenesis. Nat. Rev. Endocrinol..

[4] Guthold R., Stevens G.A., Riley L.M., Bull F.C. (2018). Worldwide trends in insufficient physical activity from 2001 to 2016: A pooled analysis of 358 population-based surveys with 1.9 million participants. Lancet Glob. Health.

[5] Dryden R., Williams B., McCowan C., Themessl-Huber M. (2012). What do we know about who does and does not attend general health checks? findings from a narrative scoping review. BMC Public Health.

[6] Nakao Y.M., Miyamoto Y., Ueshima K., Nakao K., Nakai M., Nishimura K., Yasuno S., Hosoda K., Ogawa Y., Itoh H. (2018). Effectiveness of nationwide screening and lifestyle intervention for abdominal obesity and cardiometabolic risks in Japan: The metabolic syndrome and comprehensive lifestyle intervention study on nationwide database in Japan (MetS ACTION-J study). PLoS ONE.

[7] Krogsbøll L.T., Jørgensen K.J., Grønhøj Larsen C., Gøtzsche P.C. (2012). General health checks in adults for reducing morbidity and mortality from disease: Cochrane systematic review and meta-analysis. BMJ.

[8] Krogsbøll L.T., Jørgensen K.J., Gøtzsche P.C. (2019). General health checks in adults for reducing morbidity and mortality from disease. Cochrane Database Syst. Rev..

[9] Andersen R.M. (1995). Revisiting the behavioral model and access to medical care: Does it matter?. J. Health Soc. Behav..

[10] Babitsch B., Gohl D., von Lengerke T. (2012). Re-revisiting Andersen’s Behavioral Model of Health Services Use: A systematic review of studies from 1998–2011. Psychosoc. Med..

[11] de Waard A.K.M., Wändell P.E., Holzmann M.J., Korevaar J.C., Hollander M., Gornitzki C., de Wit N.N., Schellevis F.G., Lionis C., Søndergaard J. (2018). Barriers and facilitators to participation in a health check for cardiometabolic diseases in primary care: A systematic review. Eur. J. Prev. Cardiol..

[12] Cheong A.T., Liew S.M., Khoo E.M., Mohd Zaidi N.F., Chinna K. (2017). Are interventions to increase the uptake of screening for cardiovascular disease risk factors effective? A systematic review and meta-analysis. BMC Fam. Pract..

[13] Jepson R., Clegg A., Forbes C., Lewis R., Sowden A., Kleijnen J. (2000). The determinants of screening uptake and interventions for increasing uptake: A systematic review. Health Technol. Assess.

[14] Christensen B. (1995). Payment and attendance at general practice preventive health examinations. Fam. Med..

[15] Ministry of Health, Labour and Welfare The National Health and Nutrition Survey 2018. https://www.mhlw.go.jp/stf/newpage_08789.html.

[16] Funahashi H., Nishida T., Okamura Y., Sakakibara H. (2013). Attributes of non-participants aged 40–59 years in specific health check-ups. JPN J. Public Health.

[17] Tabuchi T., Murayama H., Hoshino T., Nakayama T. (2017). An out-of-pocket cost removal intervention on fecal occult blood test attendance. Am. J. Prev. Med..

[18] Tabuchi T., Hoshino T., Nakayama T., Ito Y., Ioka A., Miyashiro I., Tsukuma H. (2013). Does removal of out-of-pocket costs for cervical and breast cancer screening work? A quasi-experimental study to evaluate the impact on attendance, attendance inequality and average cost per uptake of a Japanese government intervention. Int. J. Cancer.

[19] City of Yokohama. https://www.city.yokohama.lg.jp.

[20] Kanagawa Prefecture (2019). Analysis of Morbidity and Medical Expenditure among Local Municipalities in Kanagawa Prefecture.

[21] Franks P., Engerman J. (1991). The impact of office cholesterol testing. J. Fam. Pract..

[22] Ringel J.S., Hosek S.D., Vollaard B.A., Mahnovski S. (2002). The Elasticity of Demand for Health Care: A Review of the Literature and its Application to the Military Health System.

[23] Cheong A.T., Khoo E.M., Tong S.F., Liew S.M. (2016). To check or not to check? A qualitative study on how the public decides on health checks for cardiovascular disease prevention. PLoS ONE.

[24] Burgess C., Wright A.J., Forster A.S., Dodhia H., Miller J., Fuller F., Cajeat E., Gullford M.C. (2015). Influences on individuals’ decisions to take up the offer of a health check: A qualitative study. Health Expect.

[25] Brunner-Ziegler S., Rieder A., Stein K.V., Koppensteiner R., Hoffmann K., Dorner T.E. (2013). Predictors of participation in preventive health examinations in Austria. BMC Public Health.

[26] Hoebel J., Starker A., Jordan S., Richter M., Lampert T. (2014). Determinants of health check attendance in adults: Findings from the cross-sectional German Health Update (GEDA) study. BMC Public Health.

[27] Bjerregaard A.L., Maindal H.T., Bruun N.H., Sandbæk A. (2017). Patterns of attendance to health checks in a municipality setting: The Danish ’Check Your Health Preventive Program’. Prev. Med. Rep..

[28] Schülein S., Taylor K.J., Schriefer D., Blettner M., Klug S.J. (2017). Participation in preventive health check-ups among 19,351 women in Germany. Prev. Med. Rep..

[29] Lee Y.S., Chiu Y.L., Liao H.L., Chen J.T., Lee F.C. (2010). Factors influencing the intention to utilize out-of-pocket health checkup services: A sample of citizens from 12 townships of Taichung County in Taiwan. J. Chin. Med. Assoc..

