# Supplementary material for: Effectiveness of an Out-of-Pocket Cost Removal Intervention on Health Check Attendance in Japan
Source: Int J Environ Res Public Health. 2021 May 24;18(11):5612. doi: 10.3390/ijerph18115612 (PMC8197396; doi:10.3390/ijerph18115612)
Supplement: Supplementary file 1 [file ijerph-18-05612-s001.zip › ijerph-1223014-supplementary.pdf]

Supplementary Materials

Table S1. Increases in specific health check attendance after the out-of-pocket cost removal intervention in Aoba.

	Attendance rate	Model 1	Model 2	Model 3
		OR (95% CI)	OR (95% CI)	OR (95% CI)
Year				
2015	21.4%	0.996 (0.972–1.020)	1.011 (0.986–1.037)	1.010 (0.984–1.037)
2016	20.4%	0.938 (0.917–0.959)	0.945 (0.923–0.967)	0.949 (0.926–0.972)
2017	21.4%	1.000	1.000	1.000
2018	24.6%	1.198 (1.171–1.226)	1.184 (1.156–1.211)	1.178 (1.149–1.207)
Age				
40–49 years			0.344 (0.324–0.366)	0.345 (0.324–0.366)
50–59 years			0.470 (0.443–0.497)	0.470 (0.444–0.498)
60–69 years			0.817 (0.785–0.849)	0.817 (0.786–0.850)
70–74 years			1.000	1.000
Gender				
Male			0.778 (0.746–0.812)	0.779 (0.747–0.813)
Female			1.000	1.000
Tax exemption				
Receiving			0.915 (0.877–0.954)	0.915 (0.878–0.955)
Not receiving			1.000	1.000
Interactions				
40–49 years × year of 2015				0.959 (0.868–1.059)
50–59 years × year of 2015				1.009 (0.923–1.104)
60–69 years × year of 2015				1.034 (0.967–1.106)
40–49 years × year of 2016				1.059 (0.965–1.162)

50–59 years × year of 2016	1.018 (0.939–1.104)
60–69 years × year of 2016	1.022 (0.963–1.085)
40–49 years × year of 2018	0.952 (0.866–1.047)
50–59 years × year of 2018	1.002 (0.925–1.087)
60–69 years × year of 2018	0.992 (0.935–1.053)
Male × year of 2015	0.976 (0.923–1.032)
Male × year of 2016	0.964 (0.916–1.016)
Male × year of 2018	1.004 (0.954–1.057)
Receiving tax exemption × year of 2015	1.060 (1.003–1.120)
Receiving tax exemption × year of 2016	1.021 (0.969–1.074)
Receiving tax exemption × year of 2018	0.914 (0.869–0.963)

CI: confidence interval. OR: odds ratio.

Table S2. Increases in specific health check attendance after the out-of-pocket cost removal intervention in Kanazawa.

	Attendance rate	Model 1<br>OR (95% CI)	Model 2<br>OR (95% CI)	Model 3<br>OR (95% CI)
Year				
2015	22.3%	1.014 (0.988–1.040)	1.028 (1.001–1.056)	1.026 (0.996–1.057)
2016	21.3%	0.957 (0.934–0.981)	0.967 (0.943–0.991)	0.967 (0.940–0.994)
2017	22.1%	1.000	1.000	1.000
2018	24.7%	1.158 (1.131–1.187)	1.150 (1.122–1.178)	1.132 (1.101–1.165)
Age				
40–49 years			0.329 (0.305–0.355)	0.329 (0.305–0.354)
50–59 years			0.450 (0.419–0.482)	0.449 (0.419–0.482)
60–69 years			0.857 (0.823–0.893)	0.858 (0.824–0.894)
70–74 years			1.000	1.000
Gender				
Male			0.701 (0.669–0.735)	0.702 (0.669–0.736)
Female			1.000	1.000
Tax exemption				
Receiving			0.827 (0.789–0.867)	0.828 (0.790–0.868)
Not receiving			1.000	1.000
Interactions				
40–49 years × year of 2015				0.942 (0.833–1.064)
50–59 years × year of 2015				1.010 (0.907–1.124)
60–69 years × year of 2015				0.931 (0.868–1.001)
40–49 years × year of 2016				0.981 (0.875–1.100)
50–59 years × year of 2016				0.985 (0.890–1.090)
60–69 years × year of 2016				0.951 (0.894–1.012)

40–49 years × year of 2018	0.888 (0.790–0.998)
50–59 years × year of 2018	0.899 (0.811–0.996)
60–69 years × year of 2018	0.922 (0.868–0.980)
Male × year of 2015	1.045 (0.984–1.110)
Male × year of 2016	1.002 (0.948–1.060)
Male × year of 2018	1.026 (0.971–1.084)
Receiving tax exemption × year of 2015	1.003 (0.944–1.066)
Receiving tax exemption × year of 2016	1.004 (0.949–1.062)
Receiving tax exemption × year of 2018	0.949 (0.898–1.003)

CI: confidence interval. OR: odds ratio.

Table S3. Increases in specific health check attendance after the out-of-pocket cost removal intervention in Seya.

	Attendance rate	Model 1	Model 2	Model 3
		OR (95% CI)	OR (95% CI)	OR (95% CI)
Year				
2015	19.2%	1.034 (0.998–1.070)	1.048 (1.011–1.087)	1.038 (0.999–1.079)
2016	18.2%	0.966 (0.935–0.998)	0.976 (0.944–1.010)	0.970 (0.936–1.006)
2017	18.7%	1.000	1.000	1.000
2018	21.3%	1.178 (1.140–1.218)	1.164 (1.125–1.204)	1.152 (1.111–1.196)
Age				
40–49 years			0.291 (0.265–0.319)	0.292 (0.266–0.320)
50–59 years			0.395 (0.361–0.432)	0.396 (0.362–0.433)
60–69 years			0.764 (0.722–0.808)	0.763 (0.721–0.808)
70–74 years			1.000	1.000
Gender				
Male			0.739 (0.694–0.786)	0.739 (0.694–0.787)
Female			1.000	1.000
Tax exemption				
Receiving			0.870 (0.817–0.927)	0.870 (0.817–0.927)
Not receiving			1.000	1.000
Interactions				
40–49 years × year of 2015				0.932 (0.804–1.082)
50–59 years × year of 2015				0.968 (0.843–1.111)
60–69 years × year of 2015				1.053 (0.957–1.158)
40–49 years × year of 2016				0.981 (0.853–1.129)
50–59 years × year of 2016				0.913 (0.804–1.037)
60–69 years × year of 2016				0.997 (0.916–1.085)

40–49 years × year of 2018	0.919 (0.794–1.063)
50–59 years × year of 2018	0.880 (0.776–0.998)
60–69 years × year of 2018	0.931 (0.855–1.014)
Male × year of 2015	0.977 (0.900–1.059)
Male × year of 2016	1.006 (0.932–1.085)
Male × year of 2018	0.944 (0.874–1.019)
Receiving tax exemption × year of 2015	1.079 (0.994–1.172)
Receiving tax exemption × year of 2016	1.038 (0.961–1.120)
Receiving tax exemption × year of 2018	0.960 (0.889–1.036)

CI: confidence interval. OR: odds ratio.
